# Environmental Factors Affecting Cognitive Function among Community-Dwelling Older Adults: A Longitudinal Study

**DOI:** 10.3390/ijerph18168528

**Published:** 2021-08-12

**Authors:** Atsushi Motohiro, Takafumi Abe, Kenta Okuyama, Keiichi Onoda, Tomoko Ito, Minoru Isomura, Toru Nabika, Shunichi Kumakura

**Affiliations:** 1 Canvas Inc., 366 Satokata, Kisuki-cho, Unnnan-shi 699-1311, Shimane, Japan; 2 Department of Medical Education and Research, Faculty of Medicine, Shimane University, 89-1 Enya-cho, Izumo-shi 693-8501, Shimane, Japan; kumakura@med.shimane-u.ac.jp; 3 Center for Community-Based Healthcare Research and Education (CoHRE), Organization for Research and Academic Information, Shimane University, 223-8 Enya-cho, Izumo-shi 693-8501, Shimane, Japan; t-abe@med.shimane-u.ac.jp (T.A.); kenta.okuyama@med.lu.se (K.O.); isomura@hmn.shimane-u.ac.jp (M.I.); nabika@med.shimane-u.ac.jp (T.N.); 4 Center for Primary Health Care Research, Department of Clinical Sciences Malmö, Lund University, Jan Waldenströms gata 35, 20502 Malmö, Sweden; 5 Department of Psychology, Otemon Gakuin University, 2-1-15 Nishiai, Ibaraki City 567-8502, Osaka, Japan; onodak1@gmail.com; 6 Department of Community Health and Gerontological Nursing, Faculty of Medicine, Shimane University, 89-1 Enya-cho, Izumo 693-8501, Shimane, Japan; t-ito@med.shimane-u.ac.jp; 7 Faculty of Human Sciences, Shimane University, 1060 Nishikawatsu-cho, Matsue-shi 690-8504, Shimane, Japan; 8 Department of Functional Pathology, Faculty of Medicine, Shimane University, 89-1 Enya-cho, Izumo-shi 693-8501, Shimane, Japan

**Keywords:** cognitive function, rural mountainous area, older adults

## Abstract

Although neighborhood environmental factors have been found to be associated with cognitive decline, few longitudinal studies have focused on their effect on older adults living in rural areas. This longitudinal study aimed to investigate the role of neighborhood environmental factors in cognitive decline among rural older adults. The data of 485 older adults aged ≥60 years who were living in Unnan City in Japan and had participated in two surveys conducted between 2014 and 2018 were analyzed. Cognitive function was assessed using the Cognitive Assessment for Dementia, iPad version 2. Elevation, hilliness, residential density, and proximity to a community center were determined using geographic information system. We applied a generalized estimating equation with odds ratios (OR) and 95% confidence intervals (CIs) of cognitive decline in the quartiles of neighborhood environmental factors. A total of 56 (11.6%) participants demonstrated a decrease in cognitive function at follow up. Elevation (adjusted OR 2.58, 95% CI (1.39, 4.77) for Q4 vs. Q1) and hilliness (adjusted OR 1.93, 95% CI (1.03, 3.63) for Q4 vs. Q1) were associated with a higher likelihood of cognitive decline. The second quartiles of residential density showed significantly lower likelihoods of cognitive decline compared with the first quartiles (adjusted OR 0.36, 95% CI (0.19, 0.71) for Q2 vs. Q1). Thus, an elevated hilly environment and residential density predicted cognitive decline among rural older adults.

## 1. Introduction

Dementia is a significant public health issue in aging societies worldwide. Not only does it disrupt the lives of affected individuals and their caregivers and families, but it also imposes a significant economic burden on society [1]. Dementia affects approximately 50 million people worldwide, and this number is expected to rise to 82 million by 2030 [2].

Previous reviews have focused on the relationship between cognitive decline and personal factors such as lifestyle and health status [3,4]. In addition, air pollution, occupational exposure, and stress have been reported as physical environmental factors relevant to cognitive function [3]. Therefore, to intervene in high-risk communities for primary prevention, it is important to understand the environmental factors that predict cognitive decline.

In recent years, studies have examined the relationship between neighborhood environmental factors and health-related outcomes using a geographic information system (GIS), which has proven useful in the field of public health [5,6]. As a result, it has become clear that social cohesion, the neighborhood sidewalk environment, and proximity to nearby facilities affect cognitive function [7,8,9]. Several cross-sectional studies have also examined the relationship between various physical environmental factors and cognitive function [10,11,12]. However, there has been insufficient research on how physical environmental factors, which are unique to rural areas, affect long-term cognitive decline.

In this study, we examined the factors that contribute to cognitive decline among older adults, with the aim of developing effective measures against its progression. We focused on physical environmental factors in a rural area in Japan with a substantial aging population.

## 2. Materials and Methods

### 2.1. Design, Setting, and Sample

This longitudinal four-year follow-up study was conducted using data collected as part of the Shimane CoHRE Study in Shimane Prefecture. It targeted lifestyle-related diseases through the collection of data concerning medical history, lifestyle, and cognitive function during annual municipal health examinations [13,14,15,16]. 

**Figure 1:**
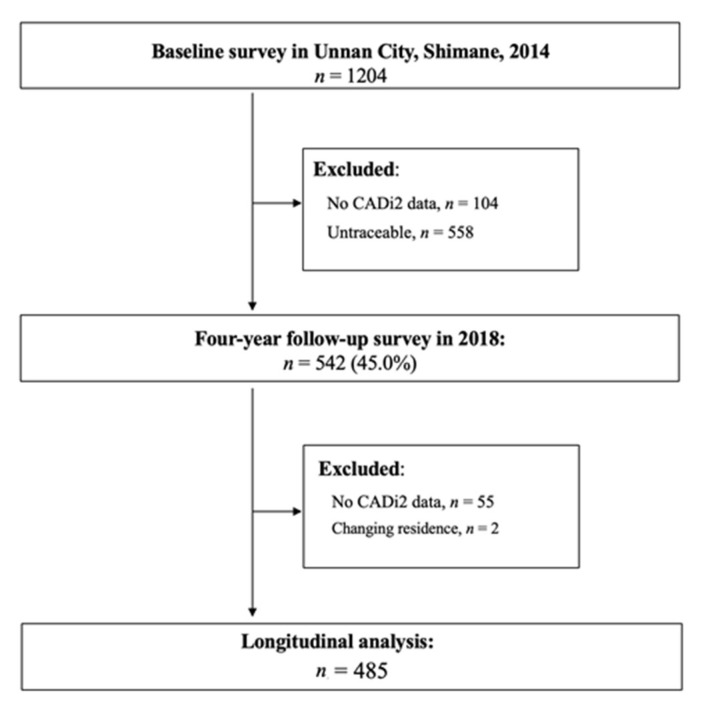
Flowchart of the study. CADi2: Cognitive Assessment for Dementia, iPad version 2.

The research team collected longitudinal data between August and October in 2014 and in 2018, in collaboration with the annual health examination program that involved the population of Unnan City, Shimane Prefecture, Japan. Annual health examinations are available once a year for residents in this municipality who are covered by the National Health Insurance. We provided information regarding this study at least once to potential participants through a document prior to conducting the health examinations. The participants were older adults aged 60 years and above who were residing in Unnan City, which has an aging population of more than 35%. The study included those who had participated in both the baseline survey in 2014 and the follow-up survey in 2018. After excluding those with missing data (*n* = 55) and those who did not reside at the same address as in 2014 (*n* = 2), the total sample size was 485 (218 males and 267 females) (Figure 1).

### 2.2. Outcome Variable

The research team assessed cognitive function using the Cognitive Assessment for Dementia, iPad version 2 (CADi2), which has been evaluated for validity and reliability [17]. The CADi2 consists of 10 items: immediate recognition, long-term memory, digits backward, orientation (month), orientation (day of the week), calculation, cube rotation, sequence making A, sequence making B, and delayed recognition. Scores ranged from 0 to 10, with higher scores indicating better cognitive function. Cognitive decline was defined using a cutoff score (<7 points) determined based on past findings; accordingly, the scores were divided into two categories. The participants used an iPad (https://apps.apple.com/jp/app/cadi2/id808586504 (accessed on 5 July 2021); currently available only in Japanese) to answer questions during an audio presentation. CADi2 data from 2014 and 2018 were used.

### 2.3. Exposure Variables

Using a GIS, we measured elevation, hilliness, residential density, and proximity to the nearest community center. We calculated hilliness and residential density within a network buffer of 1000 m from each participant’s residential address based on the actual road network [18]. Elevation was measured when the residential point fell within the 5th mesh polygon data where mean elevation value is assigned, based on the data administered and published by the Geospatial Information Authority of Japan under the Ministry of Land, Infrastructure, Transport, and Tourism. Hilliness for each individual’s network buffer was measured based on the mean land slope degree in angular units using the 5th mesh data that intersected with the network buffers. We calculated the residential density by aggregating the number of households stored by the minimum census unit as point data within the network buffer. We measured proximity to the community center based on the road network from each residential point to the nearest community center. In Japan, a community center is a public facility that can be freely used by local groups and to hold regular meetings. We categorized each calculated value into four groups using quartiles and analyzed its effect on the change in cognitive function. We conducted all spatial analyses using ArcGIS Pro.2.0 (Esri Japan). We used the most recent data available, as of 2009 for elevation and hilliness, as of 2010 for residential density, and as of 2016 for proximity to the nearest community center.

### 2.4. Covariates

Using questionnaires and empirical measurements, we recorded the following covariates: sex (male or female), age (in years), body mass index (BMI; measured height and weight data in kg/m^2^), hypertension (categorized as hypertension if any of the following conditions were met: medication for hypertension, systolic blood pressure ≥ 140 mmHg, diastolic blood pressure ≥ 90 mmHg), and depression (categorized using a cutoff score of ≥40 on the Zung Self-Rating Depression Scale) [19]. We used data of these covariates collected in 2014.

### 2.5. Statistical Analysis

We calculated descriptive statistics for all the variables for each cognitive function group (normal, declining) and examined group differences using the χ^2^ test for categorical variables.

Further, we used a generalized estimating equation with an unadjusted model (Model 1) and a model adjusted for sex, age, BMI, hypertension, depressive symptoms, and cognitive function at baseline as potential confounders (Model 2), while environmental variables were grouped by quartiles (reference group: Q1). We examined the correlations among the environmental variables and multicollinearity before applying the generalized estimating equation. Subsequently, we observed multicollinearity between each environmental variable (r ≥ 0.32, *p* < 0.001); therefore, all the environmental factors were independently included in the model for analysis.

We conducted statistical analysis using SPSS version 26.0 for Windows (IBM Corp., Armonk, NY, USA); *p*-values less than 0.05 were considered statistically significant.

### 2.6. Ethical Considerations

Data were collected after obtaining written informed consent from all participants prior to enrollment. The study received approval from the Ethics Committee of Shimane University School of Medicine (#20180420-2) and the Ethical Review Committee of the Shimane Rehabilitation Institute (#73).

## 3. Results

Table 1 presents the characteristics of the participants. There were 485 participants in this study (44.9% males and 55.1% females). Their median (interquartile range; IQR) age was 70.0 (66.0–75.0) years. Their median (IQR) BMI was 21.6 (19.9–23.8) kg/m^2^. Of all the participants, 55 (11.3%) had cognitive decline at baseline, 240 (49.5%) met the criteria for hypertension, and 91 (18.8%) had depression. As of 2018, the number of participants who demonstrated cognitive decline was 56 (11.5%).

Table 2 presents the results of the generalized estimating equation of cognitive function. In Model 2 (adjusted model), which shows the relationship between elevation and cognitive decline, there was no significant increase in cognitive decline from the first to the third quartile, but there was a significant increase in the fourth quartile (adjusted odds ratio (OR) 2.58, 95% confidence interval (CI) (1.39, 4.77)). With regard to hilliness, although there was no significant increase in cognitive decline in the second and third quartiles, it increased significantly in the fourth quartile in relation to the first quartile (adjusted OR 1.93, 95% CI (1.03, 3.63)). In terms of residential density, there was a significant decrease in the second quartile (adjusted OR 0.36, 95% CI (0.19, 0.71)). In terms of distance to the community center, there was no significant increase in cognitive decline from the second to the fourth quartile.

## 4. Discussion

In this study, we aimed to examine the effects of neighborhood environmental factors on cognitive health in order to help develop preventive strategies against cognitive decline among older adults in the community. This is the longitudinal study to focus on the hilly nature of neighborhood environmental factors that influence cognitive decline among older adults in rural Japan. The results indicate that neighborhood environment has an effect on cognitive decline. In particular, the risk of cognitive decline was higher in more hilly environments (increased elevation and hilliness). This finding is consistent with the results of a cross-sectional study conducted by Hamano et al. in a similar area [20]. It also supports the results of a longitudinal study of land slope and cognitive function by Tani et al. [9]. Moreover, we found that the second quartiles of residential density were associated with significantly lower likelihoods of cognitive decline compared with the first quartile. High residential density positively affected cognitive health; this finding is slightly different from the results of the cross-sectional study conducted by Ng et al. [11]. In that study, higher residential density tended to be associated with a lower likelihood of cognitive decline, although the results were not statistically significant. 

Several studies have investigated the relationship between cognitive function and neighborhood environment. However, most of them were cross-sectional and did not yield sufficient evidence because of the complexity of regional characteristics [21]. Our longitudinal study found the hilly nature of neighborhood environmental factors that influence cognitive decline among older adults in rural Japan. In the study by Tanaka et al., older women living in the sloped area engaged in less physical activity and had more depressive symptoms compared with their counterparts in the flat area [22]. It is possible that these personal factors cause cognitive decline in the long term [4]. Thus, the hilly environment may be one of the barriers to engagement in daily physical activity in older adults. In addition, plains tend to have a low elevation and be more densely populated because of their proximity to various living centers. Thus, we can deduce that the accessibility and availability of living centers, where people can interact with others, are more strongly related to the results as secondary psychosocial influences than the hilly environment and housing density themselves. Indeed, our study found a negative relationship between residential density and cognitive decline. Several studies have demonstrated support for the relationship between the accessibility and availability of living centers and cognitive function. A one-year follow-up study conducted in Japan found that the availability of grocery stores reduced cognitive decline [23]. In Hong Kong, a large cross-sectional study reported that library availability reduced cognitive decline [24]. Reinforcing these findings, a recent study showed that the condition of roads that affect access to living centers also affects the development of dementia [9]. In addition, populated areas may have higher social engagement than less populated areas. According to a systematic review of the literature on community and cognitive functioning, 11 out of 15 studies reported significant correlations between community engagement and various measures of cognitive functioning [21]. It has also been shown that social capital and availability of community resources have a positive impact on cognitive function [25,26]. Complementing these findings, our results suggest that there is a negative association between poor accessibility and availability of community and leisure activity centers and cognitive decline among older adults.

We focused on community centers, which are the local hubs of activity in Japan. The distance from the home to the community center can be seen as a proxy variable for social environmental factors. When we examined the relationship between cognitive function and the distance between local residences and their respective community centers, we found that participants in the second and third quartiles tended to have a lower likelihood of cognitive decline, even though the results were not statistically significant. In Japan, people can participate in cultural and educational activities at the nearest community center. Hikichi et al. [27] found that participation in a community center prevented cognitive decline, while Iwasa et al. [28] found a longitudinal inverse association between participation in hobbies and cognitive decline among community-dwelling older adults. Hence, people who live near community centers may find it easier to participate in these activities than those who live far away. In addition, walking may have affected the accessibility of community centers, since the culture in Japan is to walk rather than drive to community centers when they are relatively close by. Several studies have shown a positive relationship between residential density and walking [29,30], and it is possible that the method of travel to the community center is involved in the relationship between residential density and cognitive function. 

A few study limitations should be noted. First, some participants demonstrated improved cognitive function at follow-up. This may be attributable to preventive measures (improved diet, social interaction, etc.) taken by the participants after they were diagnosed with cognitive decline within the framework of the medical checkup. Alternatively, it may be because of the implementation of activities focused on dementia prevention in certain areas. Our study could not examine this dimension. Second, the sampling technique posed certain limitations. In contrast to past large-scale epidemiological studies, our longitudinal four-year study targeted older adults who live in mountainous regions by convenient sampling. These limit the generalization of this study within older adults who are relatively healthy enough to participate in health examinations and living in mountainous regions. Future studies need to collect larger samples from different regions by random sampling in order to improve practical and political implications. Third, we used GIS to examine the relationship between objective environmental factors and dementia, but we were not able to examine the effects of subjectively measured environmental factors (e.g., perceptions to unfavorable and disadvantaged neighborhood conditions), which have been examined in past studies [31,32]. In addition, all the environment variables were measured at one time point. Thus, we cannot discount the measurement bias related to possible longitudinal changes in neighborhood environments, such as local development, or changes in road conditions. There might have been maturation effect of repeated measurements of cognitive function (i.e., participants learn to respond on the cognitive test) as another measurement bias. Finally, we could not account for the influence of any unmeasured variables that could affect the relationship between environmental variables and cognitive decline, such as physical activity, nutritional status, educational attainment, and low-income population rate [33,34,35,36].

## 5. Conclusions

The findings of this longitudinal study suggest that elevated hilly environments increased the risk of cognitive decline among older adults in rural Japan. Moreover, residential density was negatively associated with cognitive decline. Hence, the geography surrounding residential spaces may influence cognitive function among older residents of rural areas.

## Figures and Tables

**Table 1:** Participant characteristics at baseline.

Variables	Total	Cognitive Function at Follow-Up ^a^		*p*
		Low	High	
***n***(%)	485	56 (11.5)	429(88.5)	
**Sex**				
Males	218 (44.9)	37 (66.1)	181 (42.2)	0.001
Females	267 (55.1)	19 (33.9)	248 (57.8)	
**Age, years, median (IQR)**	70.0 (66.0–75.0)	74.0 (70.0–78.0)	70.0 (66.0–74.0)	<0.001
**Body mass index, kg/m^2^, median (IQR)**	21.6 (19.9–23.8)	21.2 (19.9–22.8)	21.7 (19.9–23.9)	0.168
**Cognitive function at baseline ^a^**				
High	430 (88.7)	40 (71.4)	390 (90.9)	< 0.001
Low	55 (11.3)	16 (28.6)	39 (9.1)	
**Hypertension ^b^**				
No	245 (50.5)	21 (37.5)	224 (52.2)	0.038
Yes	240 (49.5)	35 (62.5)	205 (47.8)	
**Depressive symptoms ^c^**				
No	394 (81.2)	46 (82.1)	348 (81.1)	0.854
Yes	91 (18.8)	10 (17.9)	81 (18.9)	

^a^ Cognitive function was assessed using the Cognitive Assessment for Dementia, iPad version 2 and defined using a cutoff score of <7 points. ^b^ Hypertension was defined as follows: taking antihypertensive medication or systolic/diastolic blood pressure ≥140/90 mm Hg. ^c^ Depressive symptoms were assessed using the Zung Self-Rating Depression Scale and defined using a cutoff score of ≥40 points. IQR: interquartile range.

**Table 2:** Longitudinal associations between environmental factors and cognitive decline among older adults in rural areas.

Environmental Factors			Case/*n* (%)	Model 1 ^a^		Model 2 ^b^	
				OR	95% CI	OR	95% CI
Elevation	Low	Q1	6/121 (5.0)	1.00	(reference)	1.00	(reference)
		Q2	9/124 (7.3)	1.35	(0.68, 2.69)	1.31	(0.67, 2.56)
		Q3	15/119 (12.6)	1.69	(0.88, 3.28)	1.42	(0.73, 2.75)
	High	Q4	26/121 (21.5)	2.94	(1.57, 5.50)	2.58	(1.39, 4.77)
Hilliness	Low	Q1	9/121 (7.4)	1.00	(reference)	1.00	(reference)
		Q2	10/122 (8.2)	1.10	(0.57, 2.11)	1.32	(0.69, 2.54)
		Q3	10/121 (8.3)	1.21	(0.62, 2.36)	1.48	(0.76, 2.87)
	High	Q4	27/121 (22.3)	2.21	(1.19, 4.12)	1.93	(1.03, 3.63)
Residential density	Low	Q1	25/119 (21.0)	1.00	(reference)	1.00	(reference)
		Q2	6/123 (4.9)	0.30	(0.16, 0.57)	0.36	(0.19, 0.71)
		Q3	13/122 (10.7)	0.65	(0.37, 1.15)	0.65	(0.37, 1.15)
	High	Q4	12/121 (9.9)	0.51	(0.28, 0.93)	0.55	(0.30, 1.04)
Distance to community center	Near	Q1	16/121 (13.2)	1.00	(reference)	1.00	(reference)
		Q2	10/122 (8.2)	0.83	(0.44, 1.58)	0.86	(0.45, 1.64)
		Q3	9/121 (7.4)	0.80	(0.43, 1.47)	0.93	(0.51, 1.71)
	Far	Q4	21/121 (17.4)	1.53	(0.86, 2.72)	1.72	(0.96, 3.08)

^a^ Model 1: Crude model. ^b^ Model 2: Adjustments were made for sex, age, body mass index, hypertension, and depressive symptoms. Each environmental factor based on network buffer was analyzed separately. OR: odds ratio; CI: confidence interval.

## Data Availability

This study used data from the Shimane CoHRE study. Some data are available at the Center for Community-Based Healthcare Research and Education (CoHRE), Organization for Research and Academic Information, Shimane University, 223-8 Enya-cho, Izumo-shi, Shimane 693-8501, Japan.
